# Simulation Study of IGCT with Backside P-N Anode for Low Turn-Off Loss

**DOI:** 10.3390/mi17080923

**Published:** 2026-07-31

**Authors:** Ling Li, Shiyu Ji, Xiaoguang Wei, Yaohua Wang, Qianqian Jiao, Liang Wang, Rui Liu, Xinling Tang, Ningfei Sun, Ningyi Wang, Xinyu Huo, Yaqi Gao, Jianye Ma, Ruifeng Yue

**Affiliations:** 1 Beijing Huairou Laboratory, Beijing 101400, China; 2 The School of Integrated Circuits, Tsinghua University, Beijing 100084, China; 3 China Southern Power Grid Novel Electric Power System (BEIJING) Research Institute Co., Ltd., Beijing 100192, China

**Keywords:** power semiconductor, IGCT, low turn-off loss

## Abstract

A reverse-blocking integrated gate-commutated thyristor (RB-IGCT) incorporating a backside PN junction, referred to as PN-RBIGCT, is proposed and investigated using Sentaurus TCAD. The introduction of the PN junction establishes a reverse electric field in the anode region during turn-off, which accelerates carrier extraction and reduces switching losses. At a conduction current of 5 kA, a 40.4% reduction in turn-off energy loss (from 335.6 J to 200.0 J) is achieved, with only a moderate increase in the on-state voltage drop of the PN-RBIGCT from 1.44 V to 1.62 V.

## 1. Introduction

Power semiconductor devices play a fundamental role in modern power electronic systems, where they are extensively used for energy conversion, transmission, and regulation [1,2,3,4]. With the rapid development of renewable energy integration, flexible power transmission, and electrified transportation, the demand for devices with higher efficiency, reliability, and robustness has become increasingly urgent [5,6,7]. This has driven research and industrial interest toward advanced devices such as the integrated gate-commutated thyristor (IGCT) [8,9,10,11].

The reverse-blocking IGCT (RB-IGCT) is a fully controlled power semiconductor device with bidirectional blocking capability, proposed to address the commutation failure issue of hybrid line-commutated converters (H-LCCs) in high-voltage direct current (HVdc) transmission systems [12,13]. For instance, the world’s first hybrid commutation converter (HCC) valve based on IGCTs, developed by Tsinghua University and Beijing Huairou Laboratory, has been put into commercial operation [14]. Notably, commercial products such as Hitachi Energy’s RB-IGCT (5SHY60L6500) have been optimized for the lowest on-state voltage drop and the highest turn-off current capability [15], reflecting that the optimization targets of the device vary significantly depending on the specific application scenarios. Furthermore, in applications such as hybrid DC circuit breakers and flexible DC transmission systems that require high frequency operations, turn-off loss emerges as a critical factor limiting system performance. Therefore, this study focuses on reducing turn-off loss while maintaining adequate on-state capability, addressing the need for fast operation with low turn-off loss in these applications.

In the design of RB-IGCTs, a fundamental trade-off exists between minimizing the on-state voltage drop and reducing turn-off losses. Extremely high injection efficiency can reduce the on-state voltage and allow large current conduction, but it significantly deteriorates turn-off performance [16,17,18]. Unlike asymmetric IGCTs (AS-IGCTs), the vertical doping profile and structural constraints of RB-IGCTs prevent the direct adoption of optimization strategies such as transparent anodes or anode shorts, making their design challenges more complex [19,20].

In this paper, a backside PN junction is introduced into the conventional RB-IGCT structure. With the introduction of a PN junction at the anode side, a reverse-biased electric field can be established in the anode region during the turn-off process, which accelerates the extraction of excess electrons and enhances the electron injection efficiency of the anode junction, thereby significantly reducing the device’s turn-off losses and expanding its applicability to scenarios requiring higher turn-off frequencies. Meanwhile, the reduced hole injection efficiency leads to a moderate increase in the device’s on-state voltage drop.

## 2. Materials and Methods

This paper is conducted on an 8.5 kV/5 kA RB-IGCT [21,22]. A half-cell simulation model of the device is established using Sentaurus TCAD (V2016.06). In this model, the cathode stripe width is set to 110 μm, and the cathode mesa height is 15 μm. The total width of the half-cell is set to 250 μm. The gate–cathode region is isolated by a 1 μm thick SiO_2_ insulating layer. The thicknesses and doping concentrations of each functional region in the unit cell are summarized in Table 1.

To ensure a blocking capability of 8.5 kV, an N-type silicon substrate with a background doping concentration of 1 × 10^13^ cm^−3^ is adopted. On the back side, a P anode layer is formed by diffusion to enable reverse-blocking capability. On the front side, a deep junction diffusion process is employed to form a PN junction with a depth of 120 μm, thereby reducing the peak electric field within the device during turn-off and mitigating dynamic avalanche effects. For clarity of description, the junctions between different regions of the device are designated as follows: the junction between the P anode region and the N base region is referred to as J1, the junction between the P base region and the N base region is referred to as J2, and the junction between the N+ region and the P+ base region is referred to as J3 [23,24].

**Figure 1:**
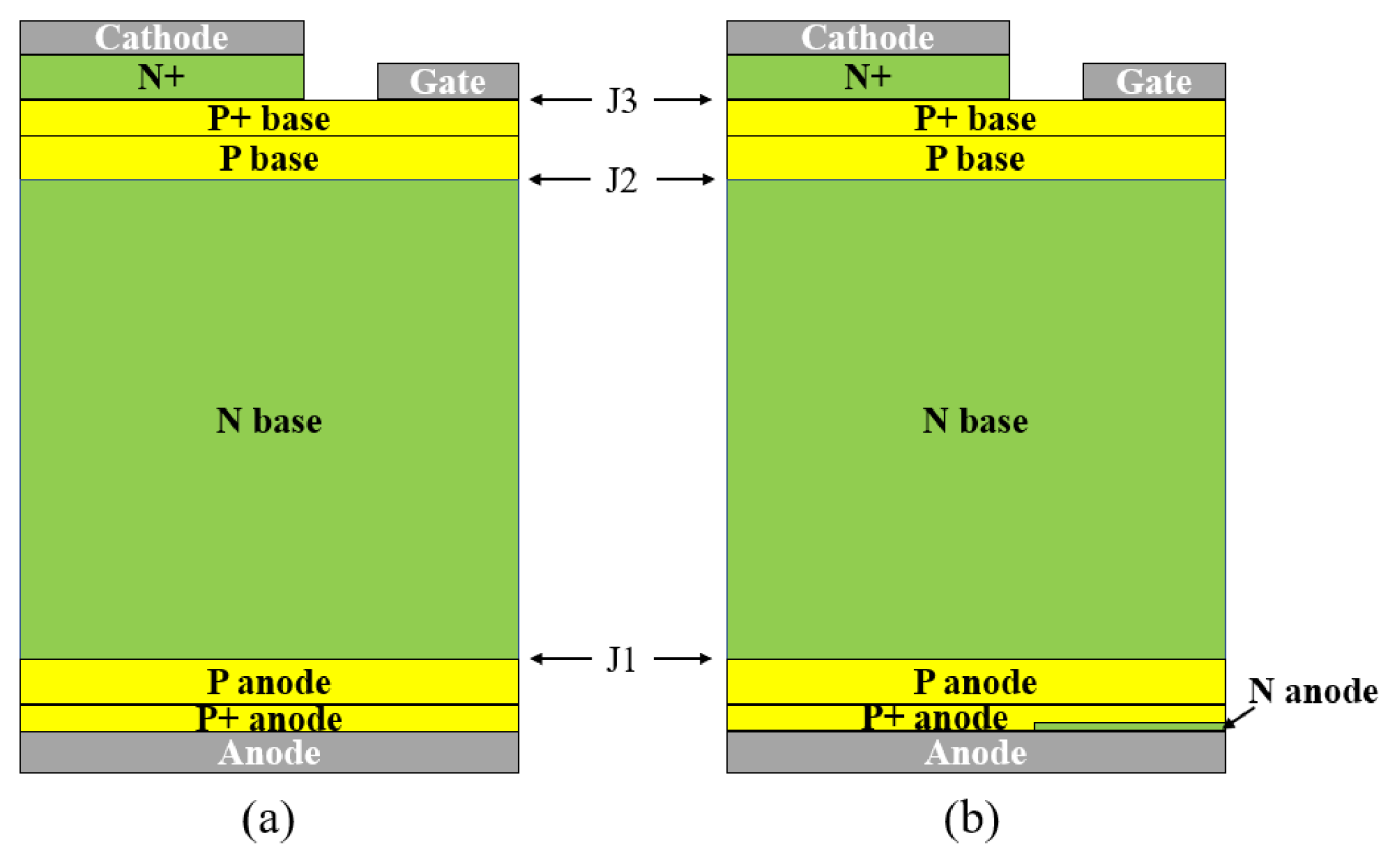
Cross-sectional schematic of a half-cell. (**a**) C-RB-IGCT. (**b**) PN-RB-IGCT.

On this basis, a thin N anode doping layer is further introduced within the P+ anode region, resulting in a novel PN-RB-IGCT structure. This modification enhances carrier extraction from the N base during turn-off, effectively lowering switching losses. A comparison between the conventional RB-IGCT (C-RB-IGCT) and the proposed PN-RB-IGCT structure is illustrated in Figure 1. The fabrication process of the PN-RB-IGCT is fully compatible with that of the C-RB-IGCT. Specifically, the front-side process flows for both devices are identical, utilizing standard techniques such as oxidation, diffusion, and lithography. The only difference lies in the backside structure: to realize the PN-RB-IGCT, a single additional ion implantation step is required on the basis of the C-RB-IGCT process. It is worth noting that this additional backside step demands relatively low process precision. Consequently, the proposed device can be reliably fabricated using mature semiconductor manufacturing technologies without introducing extra manufacturing complexity or difficulties. The detailed manufacturing process is shown in Table 2.

To accurately reflect device behavior under practical operating conditions, several physical models are incorporated into the simulation framework. For carrier transport, the Mobility model includes DopingDependence, Carrier–Carrier Scattering, High-Field Saturation, and Incomplete Ionization to account for mobility degradation under high-field and high-injection conditions [25,26,27]. The Effective Intrinsic Density model incorporates BandGapNarrowing (Slotboom) to describe the bandgap narrowing phenomenon. For recombination mechanisms, three models are employed: Shockley–Read–Hall (SRH) recombination, including DopingDependence, which represents defect-assisted recombination and is the dominant mechanism in conventional FZ-NTD silicon wafers; Auger recombination, which becomes significant under high-level injection conditions [28,29]; and Impact Ionization, modeled using the default eAvalanche and hAvalanche processes, with the critical electric field ranging from 1 × 10^5^ to 1.5 × 10^6^ V/cm, representing one of the primary causes of device failure during turn-off. Additionally, Incomplete Ionization effects are included by enabling the Incomplete Ionization model [30,31].

**Figure 2:**
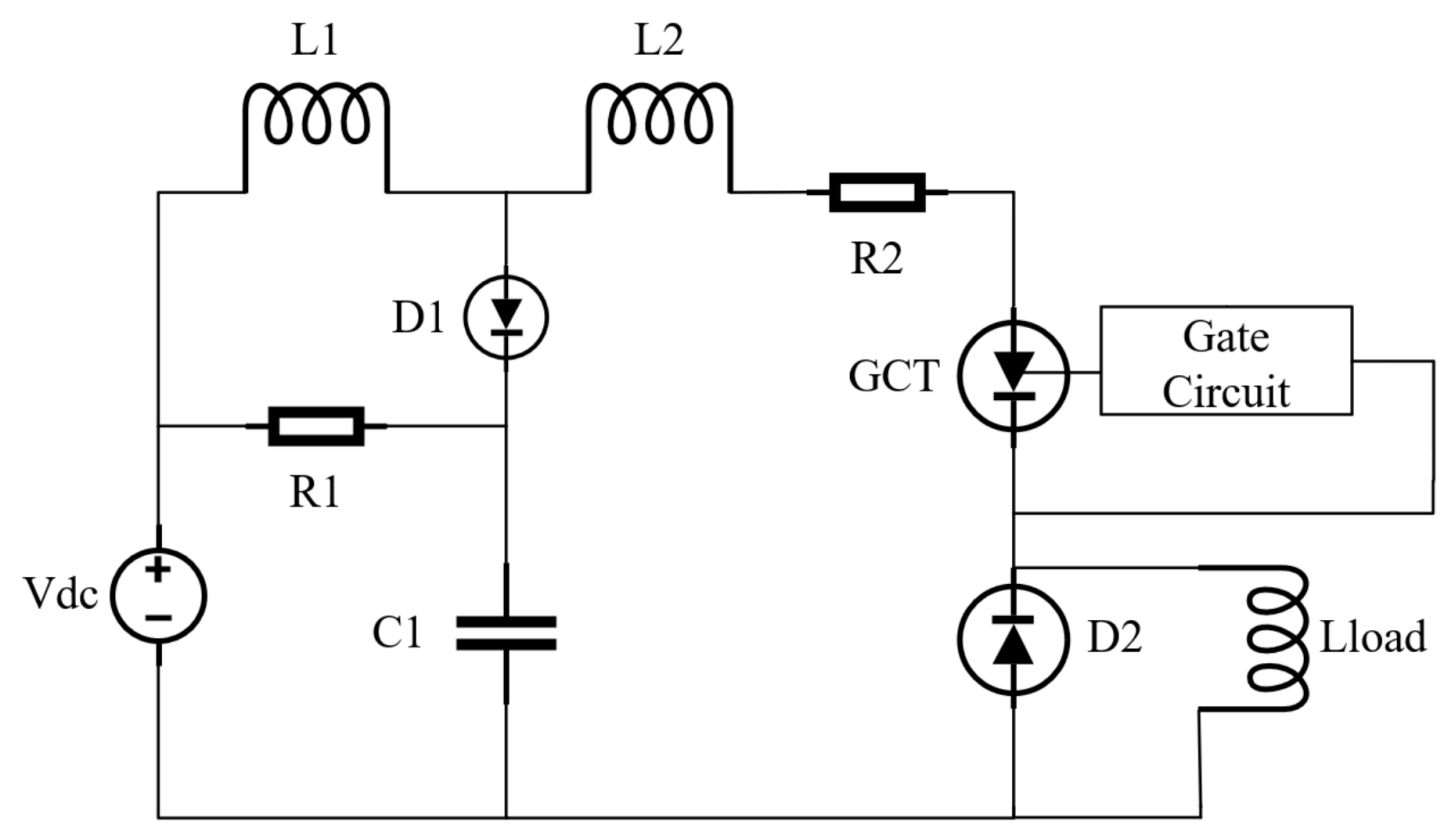
The schematic of test circuit for turn-off simulations.

The test circuit for turn-off simulations is shown in Figure 2 [32]. The parameters of this circuit are set as shown in Table 3. At the start of the test, a strong gate current pulse is applied to turn on the device. After turn-on, the anode current gradually increases, and once the current reaches the desired turn-off level, a −20 V voltage is applied across the J3 junction. This drives the J3 junction into rapid reverse bias, forcing current to commutate from the cathode to the gate and thereby enabling the device to gradually turn off. To better reflect practical conditions, both static and dynamic simulations in this study were conducted at a temperature of 115 °C.

## 3. Results and Discussion

In this section, a comparative study between the C-RB-IGCT and the proposed PN-RB-IGCT is conducted. Both static characteristics (IV curves and blocking performance) and dynamic characteristics are analyzed, with a detailed discussion of the turn-off transient process.

### 3.1. Static Characteristics

**Figure 3:**
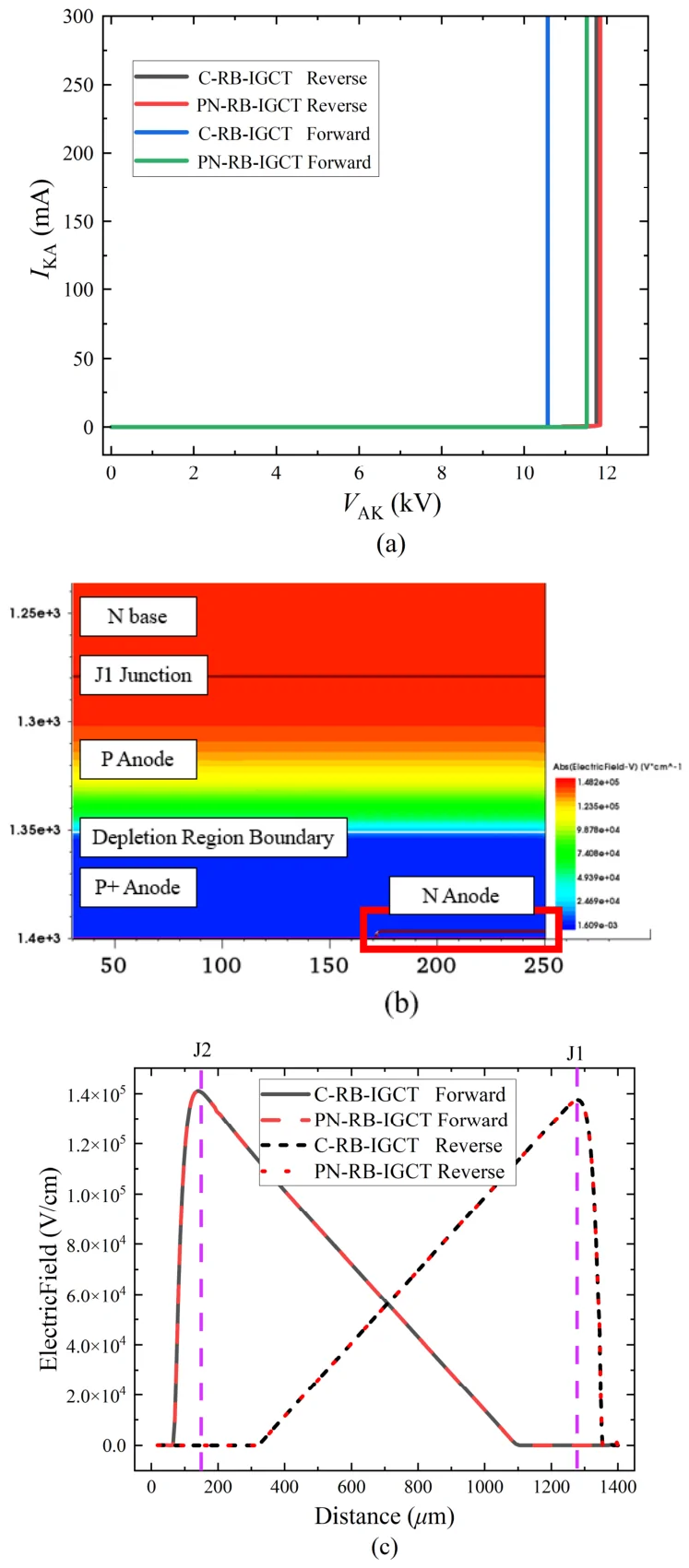
Electrical characteristics of C-RB-IGCT and PN-RB-IGCT. (**a**) Forward-blocking I–V characteristics. (**b**) The electric field distribution near the J1 junction under reverse-blocking conditions. (**c**) Vertical electric field distribution at 8.5 kV. The vertical pink dashed lines denote the positions of the J1 and J2 junctions.

Since the RB-IGCT is a bidirectional blocking device, P-type regions are formed on both the front- and backside surfaces by diffusion processes to sustain the blocking voltage under reverse and forward bias. Meanwhile, when simulating the blocking performance of the device, it is necessary to configure a gate–cathode short circuit. The simulated forward- and reverse-blocking I–V characteristics and electric field distributions of the two devices are presented in Figure 3. The calculation results show that the forward- and reverse-blocking voltages of the C-RB-IGCT are 10.56 kV and 11.74kV, respectively, while those of the PN-RB-IGCT are 11.51 kV and 11.76 kV, respectively. Since the front and back sides of the device adopt essentially symmetric P doping profiles, the forward- and reverse-blocking voltages are nearly identical. Although a PN junction is introduced on the back side of the PN-RB-IGCT, its N region is relatively thin and does not extend into the depletion region, as shown in Figure 3b. Therefore, it does not reduce the reverse-blocking capability of the device. The forward- and reverse-blocking electric fields at 8.5 kV are shown in Figure 3c. Analysis of the electric field distribution further indicates that both devices exhibit similar characteristics under blocking conditions: in forward blocking, the peak electric field is located near the J2 junction, whereas in reverse blocking, the peak field appears at the J1 junction. In both cases, the electric field profiles exhibit a triangular distribution, confirming that the introduction of the anode PN junction does not alter the fundamental blocking mechanism of the device.

**Figure 4:**
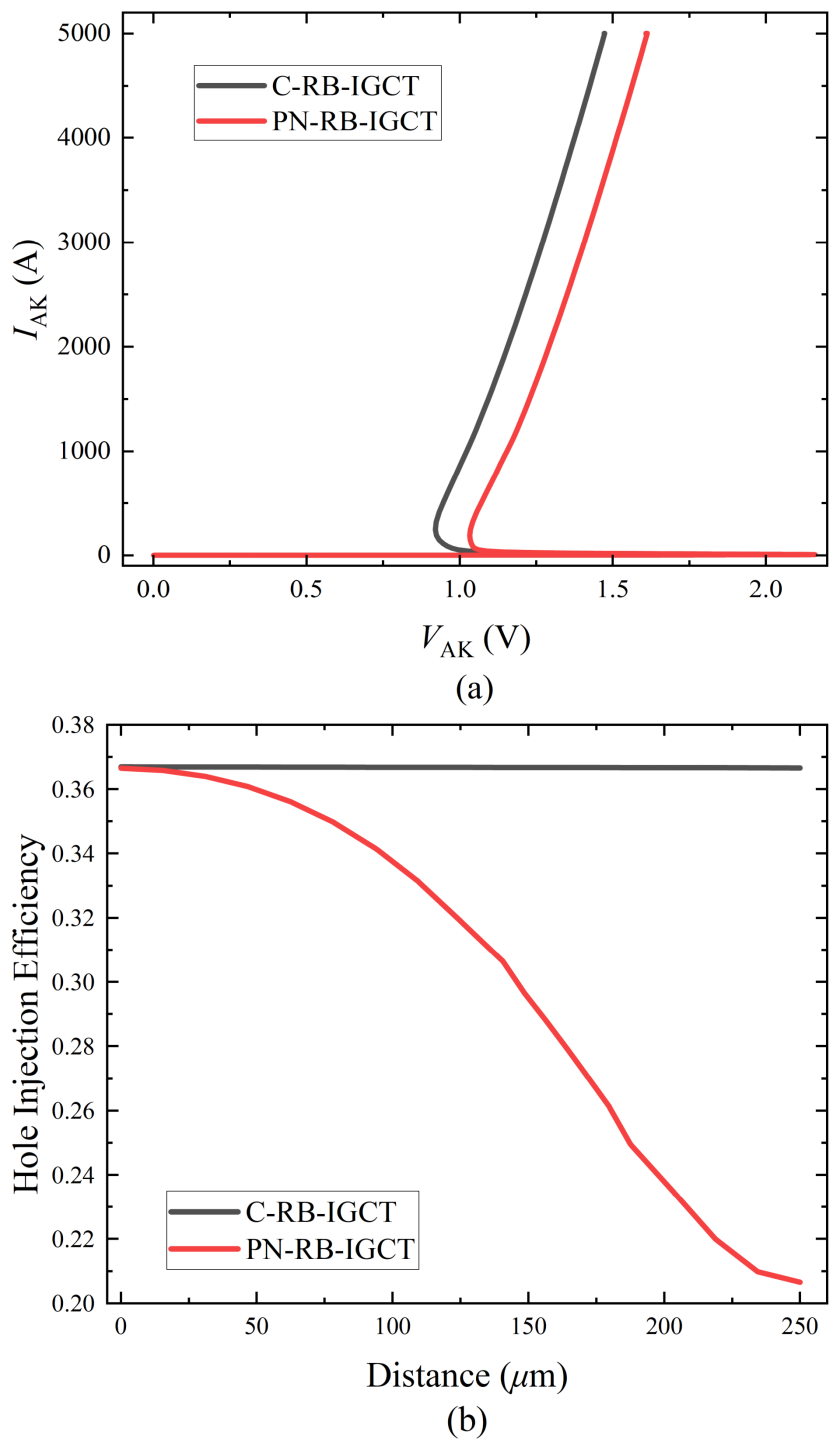
(**a**) Forward conduction IV characteristics of C-RB-IGCT and PN-RB-IGCT. (**b**) Hole injection efficiency at the J1 junction.

Figure 4a illustrates the forward conduction I-V characteristics of the two devices. Under the same current condition (*I*_AK_ = 5 kA), the on-state voltage drop of the C-RB-IGCT is 1.44 V, while that of the PN-RB-IGCT increases to 1.62 V, representing an increment of 12.5%. By examining the hole current at the J1 junction at this operating point, as shown in Figure 4b, it can be observed that the introduction of the backside PN junction reduces the hole injection efficiency. This reduction weakens the conductivity modulation effect, thereby leading to a slight increase in the forward on-state voltage drop.

### 3.2. Turn-Off Characteristics

The turn-off performance was evaluated using the test circuit shown in Figure 2. The device was first turned on by applying a strong gate current pulse. After turn-on, the anode current gradually increased, with the load inductance *L*_load_ controlling the current rise rate. Once the anode current reached the desired turn-off level of 5 kA, a −20 V voltage was applied across the J3 junction, driving it into rapid reverse bias to force current commutation from the cathode to the gate and thereby enable the device to turn off gradually.

**Figure 5:**
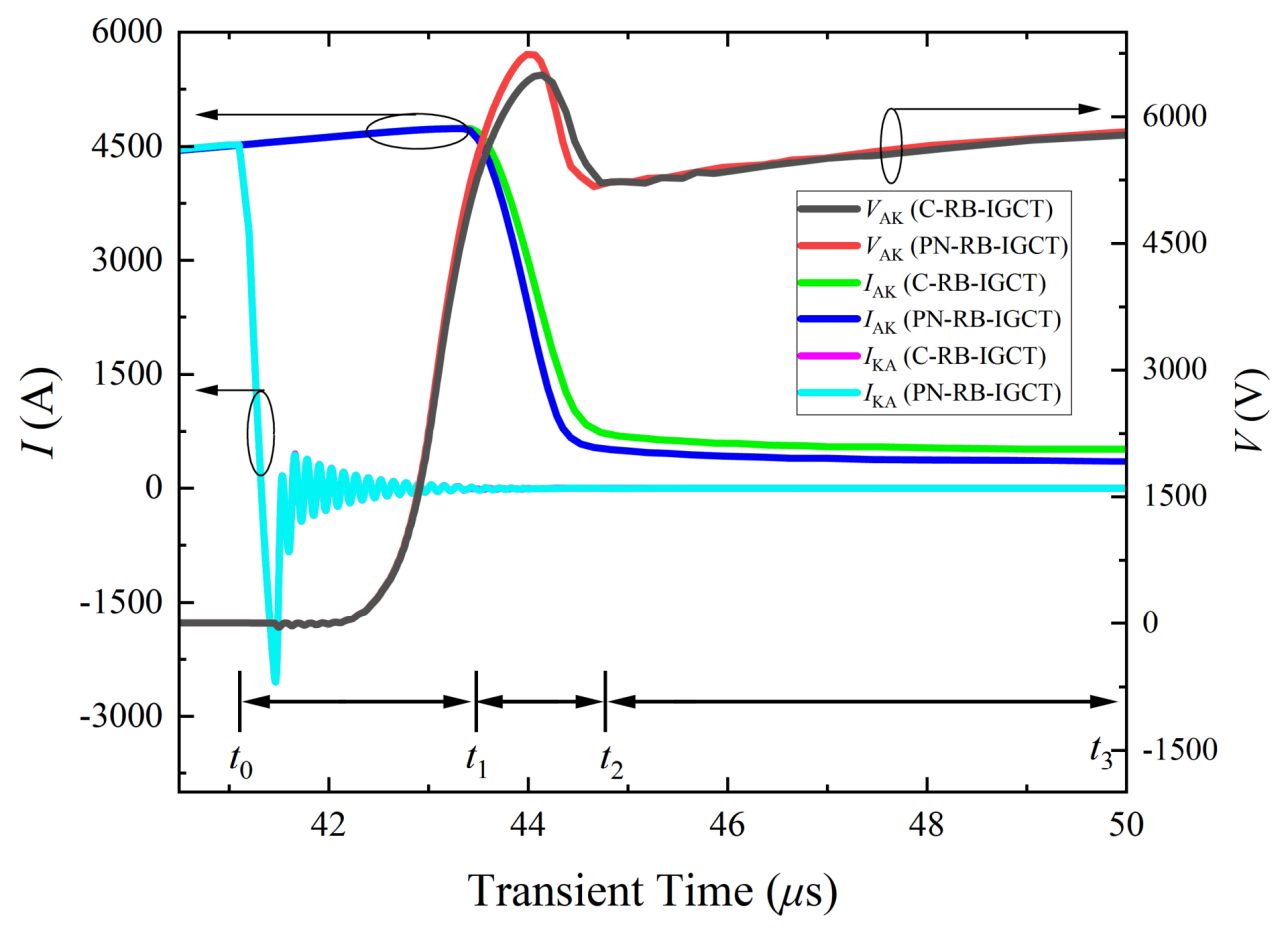
Turn-off characteristic curves of the C-RB-IGCT and PN-RB-IGCT.

Figure 5 presents the simulated turn-off characteristic curves of the two devices. Typically, the turn-off process of IGCTs can be divided into three distinct stages: the delay stage (*t*_0_ − *t*_1_), the current fall stage *(t*_1_ − *t*_2_), and the current tail stage (*t*_2_ − *t*_3_).

**Figure 6:**
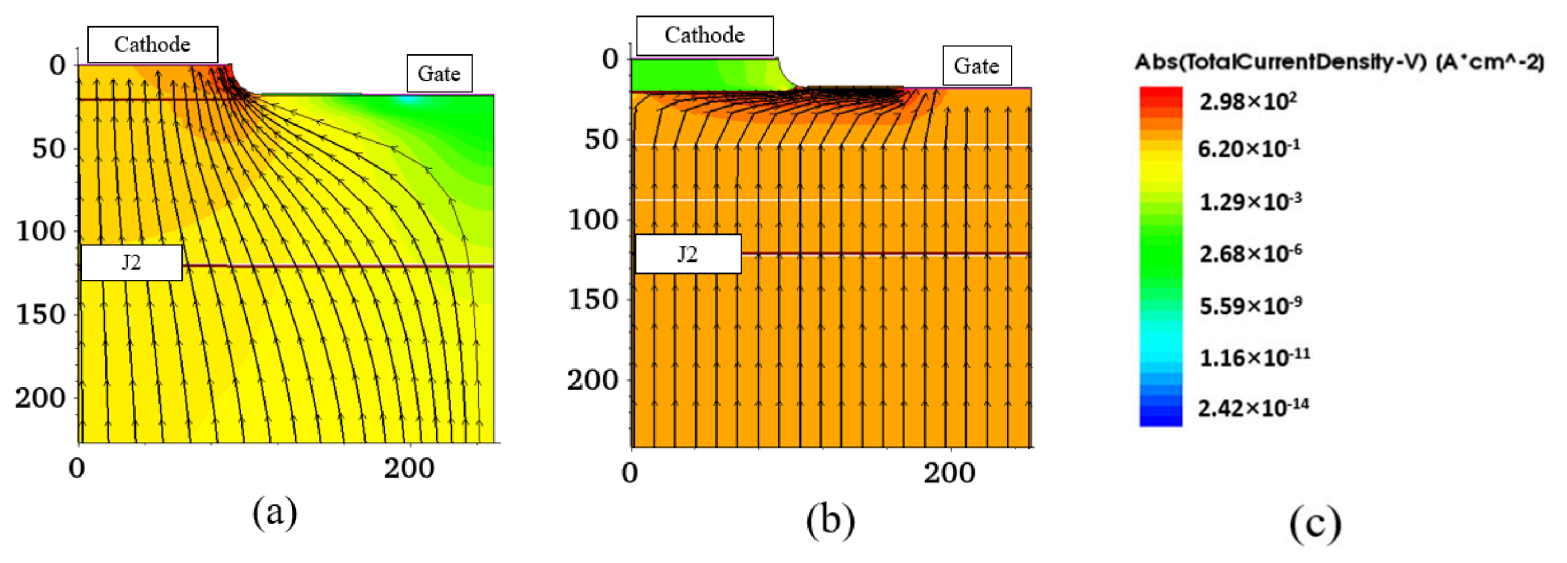
(**a**) Current distribution of the IGCT device in the conducting state. (**b**) Current distribution of the IGCT device during the commutation process. (**c**) The color scale legend. The arrows indicate the direction of current flow.

During the delay stage, a −20 V voltage is applied between the gate and cathode, which rapidly blocks the J3 junction and redirects the current to flow through the gate terminal, as shown in Figure 6a,b. It can be observed from the interval *t*_0_ − *t*_1_ of Figure 5 that the cathode current (*I*_KA_) drops to zero within a very short time, indicating that the device has been successfully turned off. At the same time, the anode current (*I*_AK_) remains nearly unchanged, indicating that current commutation to the gate has been completed. After commutation, a transient increase in cathode current occurs, caused by the reverse recovery of a large number of excess electrons stored in the N+ cathode region.

Since the gate and cathode doping profilers of the two devices are identical, it can be observed from Figure 5 that their behaviors during the delay stage are essentially the same. In the current fall stage, the anode current (*I*_AK_) begins to decrease, corresponding to the interval *t*_1_ − *t*_2_ in Figure 5. During this stage, both the gate and anode continue to extract excess carriers, leading to the expansion of the depletion region around the J2 junction and the progressive extension of the electric field toward the anode side. Consequently, the anode voltage rises toward the DC bus voltage. Excess holes are swept into the P base region through the reverse-biased J2 junction and extracted via the gate, while excess electrons are injected into the anode region through J1.

**Figure 7:**
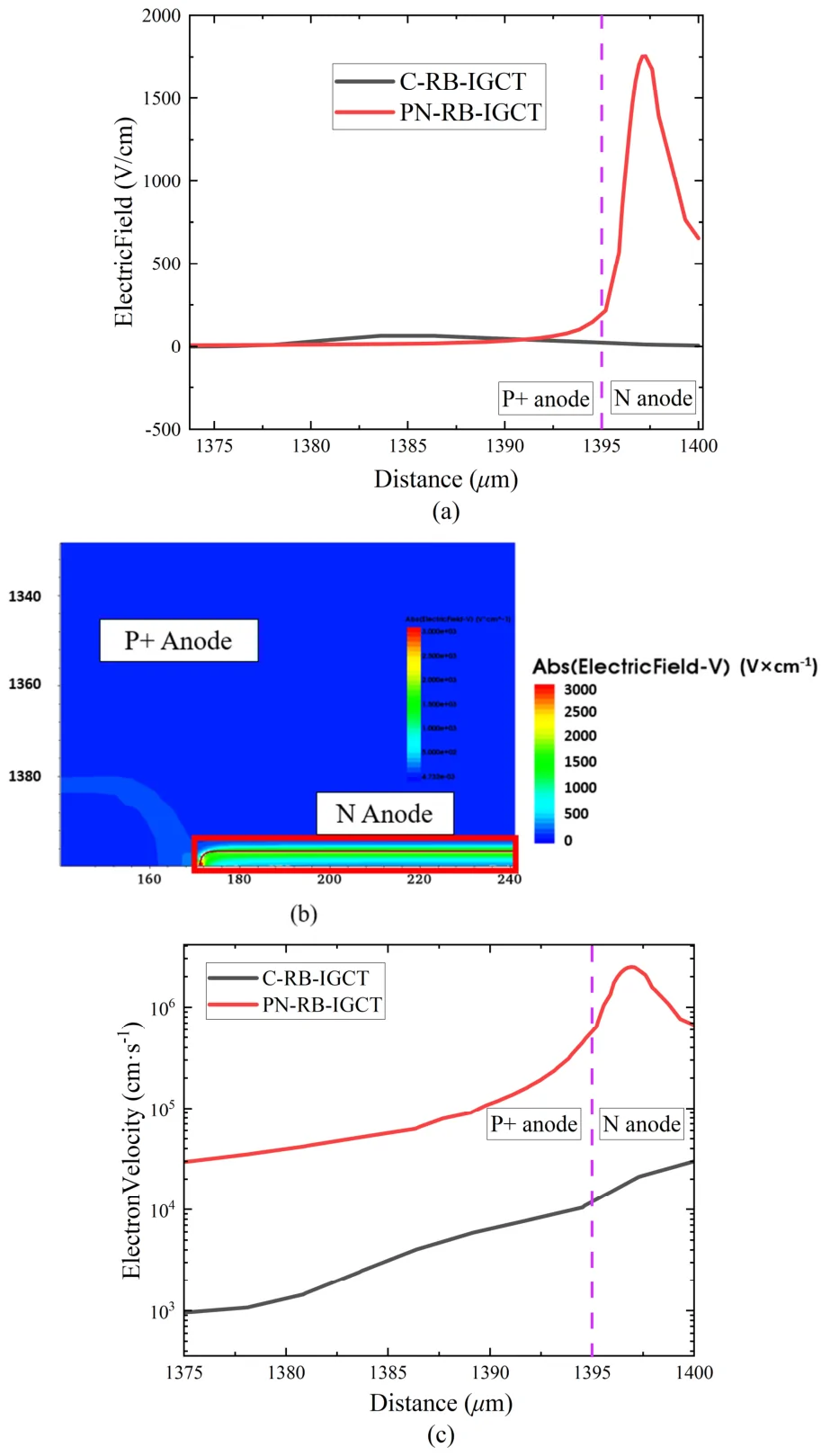
(**a**) Electric field distribution in the P+ anode region of C-RB-IGCT and PN-RB-IGCT during turn-off. (**b**) Electric field distribution near the anode PN junction during turn-off. (**c**) Electron velocity in the P+ anode region of C-RB-IGCT and PN-RB-IGCT during turn-off. The red box and pink dashed line denote the positions of the backside PN junctions.

In the current fall stage, the PN-RB-IGCT exhibits a higher current decay rate than the C-RB-IGCT, and it also shows a smaller tail current. The PN-RB-IGCT benefits from the additional PN structure at the anode, which establishes a reverse electric field during turn-off, as illustrated in Figure 7a,b. This field significantly increases the electron velocity near the anode, as shown in Figure 7c, and provides an efficient extraction path for electrons injected into the P+ anode region, thereby improving electron injection efficiency and accelerating the current fall process.

**Figure 8:**
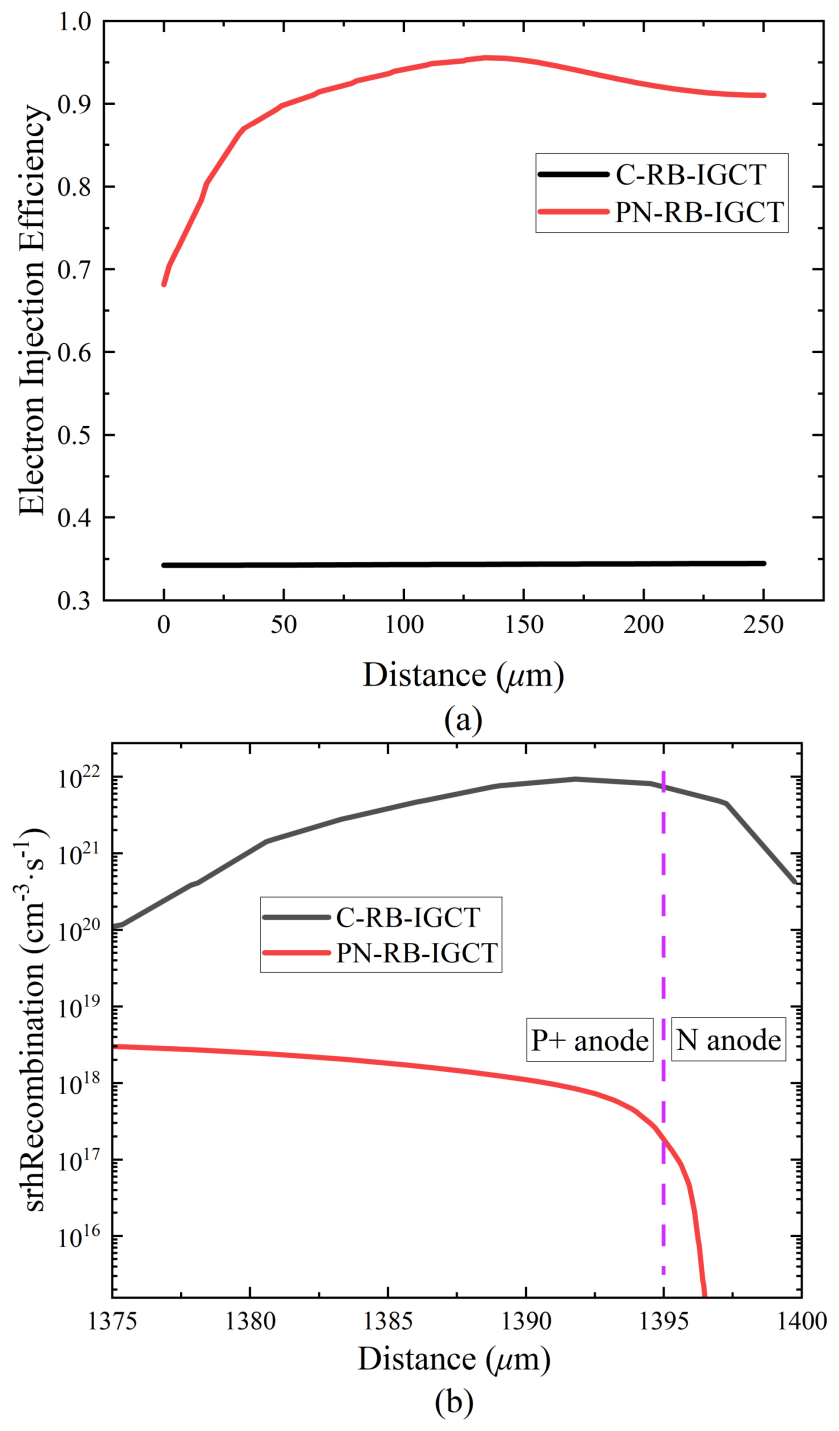
(**a**) Electron injection efficiency at the J1 junction during the current tail stage. (**b**) Calculated SRH recombination results in the P region during the current tail stage. The pink dashed line denotes the positions of the backside PN junctions.

During the current tail stage, the remaining non-equilibrium carriers inside the device continue to be extracted through the gate and the anode, or are removed via recombination, until the anode current approaches zero and the device is fully turned off. Figure 8a,b show the electron injection efficiency at the J1 junction and the SRH recombination near the anode PN junction during the current tail stage, respectively. For the C-RB-IGCT, the high hole concentration in the anode P+ region results in reduced electron injection efficiency at J1 and many of the injected electrons recombine within the anode base, causing significant turn-off energy loss.

In the PN-RB-IGCT, during the turn-off transient, as the anode voltage increases, the introduced backside PN junction enters a reverse-biased state. The electric field established by this reverse-biased PN structure actively accelerates the transit of electrons crossing the J1 junction, enabling them to be rapidly swept into the anode. Consequently, this mechanism significantly enhances the electron injection efficiency of the J1 junction and reduces SRH recombination. As a result, the remaining carriers are extracted more rapidly, ultimately leading to a shorter turn-off time and lower turn-off losses.

The turn-off energy losses of the two devices were calculated by integrating the product of voltage and current over time, yielding results of 335.6 J and 200.0 J, respectively. The results demonstrate that the PN-RB-IGCT achieves a much shorter turn-off time and significantly lower turn-off energy losses. Specifically, the turn-off energy loss is reduced from 335.6 J to 200.0 J, representing a reduction of 40.4%.

## 4. Conclusions

A novel PN-RB-IGCT structure has been proposed and analyzed through TCAD simulations. Compared with the conventional RB-IGCT, the PN-RB-IGCT demonstrates a significantly enhanced 40.4% reduction in turn-off energy loss, while maintaining nearly unchanged reverse-blocking performance. The on-state voltage drop increases moderately due to reduced hole injection efficiency. However, the significantly lower turn-off loss allows the device to operate at much higher switching frequencies. Therefore, the proposed structure is highly advantageous in higher-frequency applications. These results confirm that introducing a backside PN junction at the anode is an effective strategy to improve the performance of RB-IGCTs, offering valuable design guidance for the development of future high-power semiconductor devices.

## Figures and Tables

**Table 1:** Doping concentrations of each functional region.

Doping Area	Peak Value (cm^−3^)	Doping Depth (μm)
N+	1 × 10^20^	15
P+ base	5 × 10^17^	60
P base	8 × 10^14^	120
P anode	8 × 10^14^	120
P+ anode	1 × 10^18^	60
N anode	1 × 10^15^	5

**Table 2:** Manufacturing process of PN-RB-IGCT.

No.	Process Step
1	P base and P+ anode region doping and diffusion using aluminum
2	P+ base and P+ anode region doping and diffusion using boron
3	N+ pre-doping using phosphorus
4	Cathode mesa etching
5	N+ region diffusion
6	Backside lithography to protect the P+ anode region, and ion implantation of phosphorus (P) for the N anode region diffusion
7	SiO_2_ passivation layer etching
8	Gate, cathode, and anode metallization

**Table 3:** Table of test circuit parameters.

Component		Parameter
C1		20 μF
R1		0.4 Ω
R2		0.03 Ω
L1		3 μH
L2		0.3 μH
Lload		40 μH
T		388 K
Vdc		5000 V
D1, D2 (SPICE Model)	Transit time	50 ns
	Reverse breakdown voltage	20 kV

## Data Availability

The original contributions presented in this study are included in the article. Further inquiries can be directed to the corresponding author.
